# Rapid and Highly Sensitive Detection of Malaria-Infected Erythrocytes Using a Cell Microarray Chip

**DOI:** 10.1371/journal.pone.0013179

**Published:** 2010-10-13

**Authors:** Shouki Yatsushiro, Shohei Yamamura, Yuka Yamaguchi, Yasuo Shinohara, Eiichi Tamiya, Toshihiro Horii, Yoshinobu Baba, Masatoshi Kataoka

**Affiliations:** 1 Health Technology Research Center, National Institute of Advanced Industrial Science and Technology (AIST), Takamatsu, Japan; 2 Department of Molecular and Pharmaceutical Biotechnology, Graduate School of Pharmaceutical Sciences, University of Tokushima, Tokushima, Japan; 3 Division of Protein Expression, Institute for Genome Research, University of Tokushima, Tokushima, Japan; 4 Department of Applied Physics, Graduate School of Engineering, Osaka University, Suita, Japan; 5 Department of Molecular Protozoology, Research Institute for Microbial Diseases, Osaka University, Suita, Japan; 6 Department of Applied Chemistry, Graduate School of Engineering, Nagoya University, Nagoya, Japan; BMSI-A*STAR, Singapore

## Abstract

**Background:**

Malaria is one of the major human infectious diseases in many endemic countries. For prevention of the spread of malaria, it is necessary to develop an early, sensitive, accurate and conventional diagnosis system.

**Methods and Findings:**

A cell microarray chip was used to detect for malaria-infected erythrocytes. The chip, with 20,944 microchambers (105 µm width and 50 µm depth), was made from polystyrene, and the formation of monolayers of erythrocytes in the microchambers was observed. Cultured *Plasmodium falciparum* strain 3D7 was used to examine the potential of the cell microarray chip for malaria diagnosis. An erythrocyte suspension in a nuclear staining dye, SYTO 59, was dispersed on the chip surface, followed by 10 min standing to allow the erythrocytes to settle down into the microchambers. About 130 erythrocytes were accommodated in each microchamber, there being over 2,700,000 erythrocytes in total on a chip. A microarray scanner was employed to detect any fluorescence-positive erythrocytes within 5 min, and 0.0001% parasitemia could be detected. To examine the contamination by leukocytes of purified erythrocytes from human blood, 20 µl of whole blood was mixed with 10 ml of RPMI 1640, and the mixture was passed through a leukocyte isolation filter. The eluted portion was centrifuged at 1,000×*g* for 2 min, and the pellet was dispersed in 1.0 ml of medium. SYTO 59 was added to the erythrocyte suspension, followed by analysis on a cell microarray chip. Similar accommodation of cells in the microchambers was observed. The number of contaminating leukocytes was less than 1 on a cell microarray chip.

**Conclusion:**

The potential of the cell microarray chip for the detection of malaria-infected erythrocytes was shown, it offering 10–100 times higher sensitivity than that of conventional light microscopy and easy operation in 15 min with purified erythrocytes.

## Introduction

Malaria is one of the major human infectious diseases in over 100 endemic countries, there being approximately 300 million clinical cases and 2 million fatalities per year [1]. Prompt and accurate diagnosis is one of the keys for effective disease management, being one of the main interventions of the global malaria control strategy [2]. Conventional light microscopy is widely used for the detection and quantification of malaria parasites, and is recognized as the gold standard. In most settings, the procedure consists of: collecting a finger-prick blood sample; preparing thin and thick blood smears; staining the smears with Giemsa; and examining the smears under a microscope for the presence of malaria parasites in the erythrocytes [3]. However, this microscopic detection method is exacting and depends on a good staining technique and well supervised technicians. Milne *et al.* found that most routine diagnostic laboratories generally achieved low detection sensitivity (average, 0.01% parasitemia) on examination of the results from British laboratories submitted to the Malaria Reference Laboratory [4]. Even with excellent erythrocyte preparation and good technicians, the detection limit is low (0.001% parasitemia) and approximately 1 hr is required for the detection of a sufficient number of infected erythrocytes [5], [6]. So, it is quite difficult to detect malaria infection before the appearance of severe symptoms including high fever. Although immunochromatography was recently developed for malaria detection with easy operation and a rapid detection time (20 min), the detection limit is similar to that of microscopy observation with Giemsa staining [7], [8]. Although several new methods of malaria diagnosis based on flow cytometry or real-time PCR have been developed [9]–[12], some disadvantages remain, i.e., the relatively low detection limit for flow cytometry and the requirement of several hours for the detection of malaria parasites by real-time PCR. For prevention of the spread of malaria in the world, it is necessary to develop an early, sensitive, accurate and convenient diagnosis system [2].

**Figure 1:**
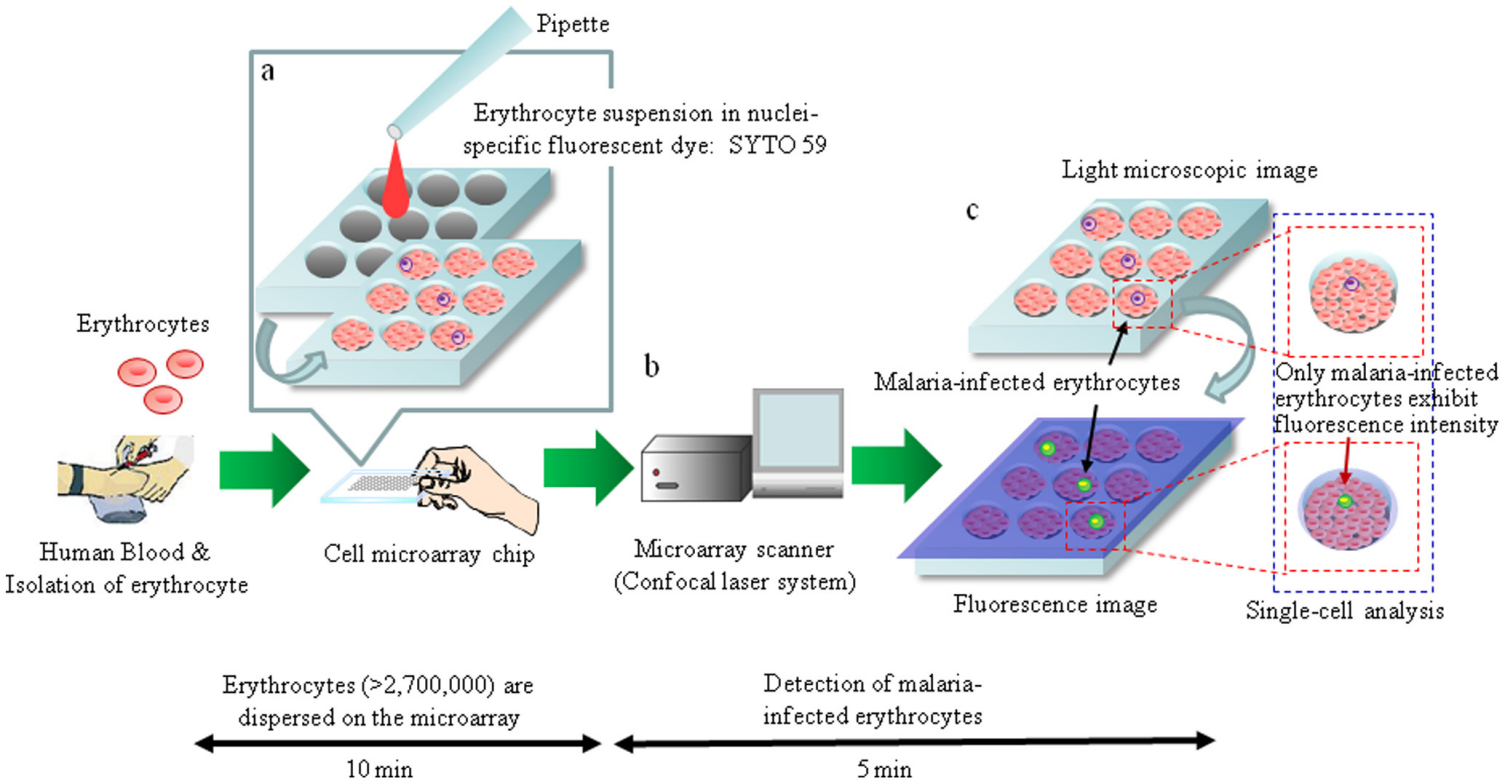
Schematic process for detection of malaria-infected erythrocytes on a cell microarray chip. (**a**) Erythrocytes stained with a nuclei-specific fluorescent dye, SYTO 59, for the staining of malaria nuclei were dispersed on a cell microarray chip using a pipette, which led to the formation of a monolayer of erythrocytes in the microchambers. (**b**) Malaria-infected erythrocytes were detected using a microarray scanner with a confocal fluorescence laser by monitoring fluorescence-positive erythrocytes. (**c**) The target malaria-infected erythrocytes were analyzed quantitatively at the single-cell level.

Recently, microchip technologies have been expected to allow high-throughput and highly sensitive analysis of the functions of individual cells [13]. In our previous study, we developed a single-cell microarray chip for the analysis of antigen-specific single B-cells [14]. Recently, Jin *et al.* improved this single-cell microarray chip and developed a new system that can directly detect the secretion of antibodies by single cells [15]. Here, we have developed a novel high-throughput screening and analysis system for malaria-infected erythrocytes allowing ultra-high sensitivity in a short time involving a cell microarray chip made from polystyrene with over 20,000 individually addressable microchambers. Our cell microarray chip has been improved to allow the regular dispersion of an erythrocyte suspension in a nuclear staining fluorescence dye in the microchambers, with the formation of a monolayer, and analysis with a microarray scanner for detection of the presence of fluorescence-positive nuclei in erythrocytes (Fig. 1a-c). The potential of a cell microarray chip system for the early diagnosis of malaria was shown, it allowing ultra-highly sensitive and accurate detection of malaria-infected erythrocytes in a short time.

## Methods

### Construction of a cell microarray chip

**Figure 2:**
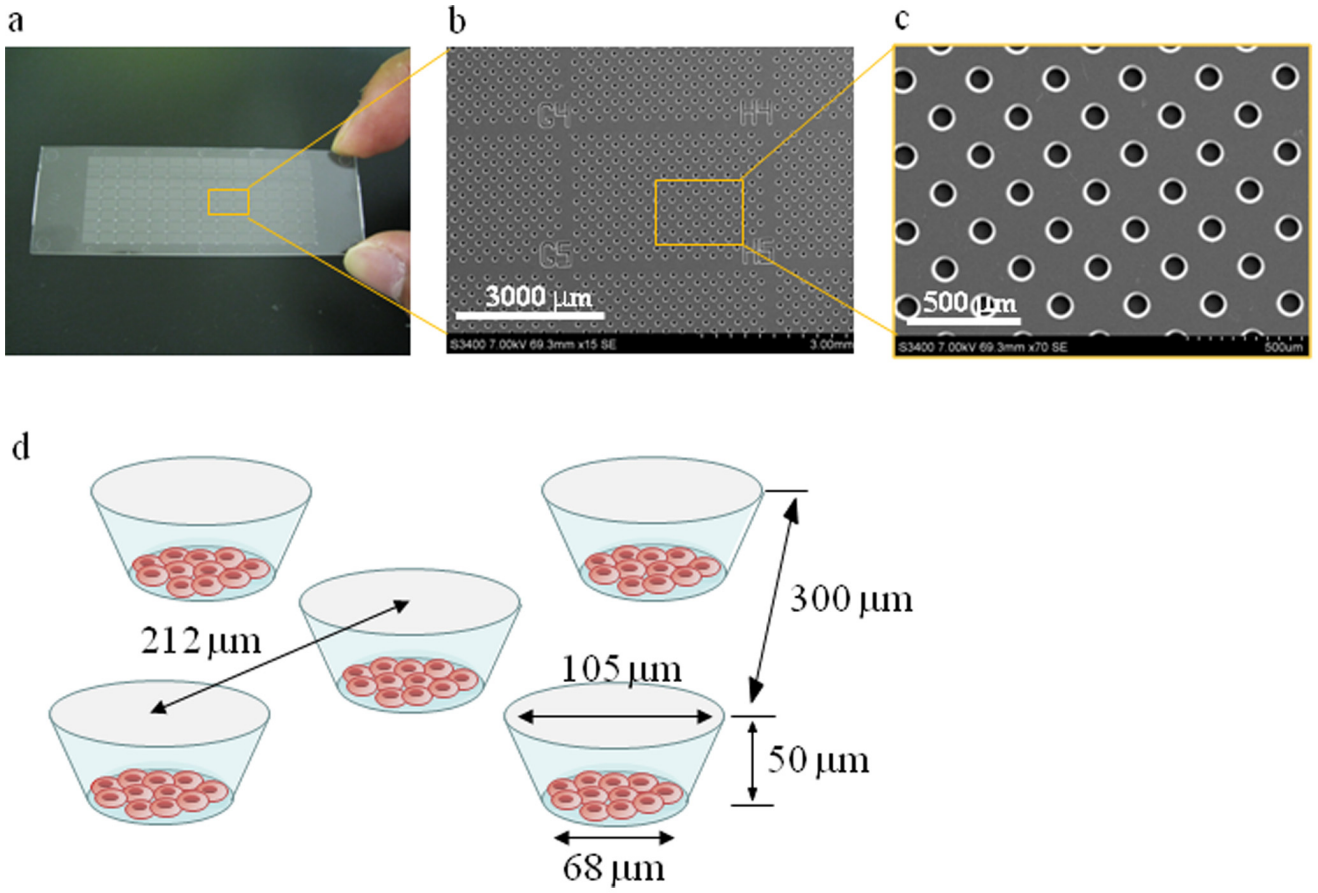
Construction of a cell microarray chip. (**a**) A real picture and (**b**, **c**) SEM images of a cell microarray chip. The microarray chip comprises 20,944 microchambers in a plastic slide of slide glass size. The microarray chip has 112 (14×8) clusters of 187 microchambers. (**d**) Each microchamber is 105 µm in width, 50 µm in depth and 300 µm in pitch, and comprises a frustum with a 68 µm diameter flat bottom for the accommodation of erythrocytes as a monolayer.

A cell microarray chip with 20,944 microchambers (105 µm width, 50 µm depth, and 300 µm pitch) was made from polystyrene by the Lithographie Galvanoformung Abformung process by Starlight Co. Ltd. (Osaka, Japan) (Fig. 2a-c) [14]. The polystyrene microarray chip was fabricated by injection molding with a nickel mold. The microarray chip has 112 (14×8) clusters of 187 microchambers, respectively. There are block numbers on the clusters for easy confirmation of malaria-infected erythrocytes. Each microchamber comprises a frustum in shape (Fig. 2d). The cell microarray chip surface is rendered hydrophilic by means of reactive ion-etching treatment using a SAMCO RIE system (SAMCO, Inc., Tokyo, Japan) to achieve erythrocyte confinement in the microchambers. The effect of reactive ion-etching exposure on the microarray chip surface was examined by measuring the contact angle of water on the chip surface using a contact-angle meter (Kyowa Interface Science Co., Ltd., Saitama, Japan) [14].

### Malaria culture

*P. falciparum* strain 3D7 cells were cultured in RPMI 1640 medium (Nacalai Tesque, Inc., Tokyo, Japan) containing 50 µg/ml gentamycin (SIGMA-Aldrich, Co., MO) and 10% O^+^ human serum at a hematocrit of 5%, according to the established method of Trager and Jensen [16]. Human blood was centrifuged at 1,000×*g* for 10 min and then washed three times with culture medium to obtain human erythrocytes. The supernatant and buffy coat were carefully removed by aspiration after washing, and the leukocytes remaining among the washed erythrocytes were removed with a leukocyte isolation filter, LeukoLOCK™ (Ambion, Inc., TX), under gravitational force. It required less than 1 min for this isolation of leukocytes with LeukoLOCK™. The purified erythrocytes were employed for the malaria culture. The parasite concentration (parasitemia) was calculated by determining the number of malaria-infected erythrocytes among 30,000 erythrocytes and expressed as the ratio to non-malaria-infected erythrocytes. Erythrocytes exhibiting 0.1% parasitemia were added to each plate in 10 ml of culture medium to give a final hematocrit of 3%. The plates were incubated at 37°C under 5% CO_2_, 5% O_2_, and 90% N_2_ gas.

### Microscopy and Giemsa staining

Two microliters of a malaria culture was smeared so as to produce a thin film on a slide. Each slide was stained with 5% Giemsa (Merck, Co., Ltd., Germany) stain in phosphate-buffered saline (pH 7.2), and then examined under a light microscope (Olympus, Co., Ltd., Tokyo, Japan), at a magnification of ×1,000, for the presence or absence of malaria parasites [3]. The cell microarray chip is similar in size and surface condition to a slide glass. Thus, it is possible that the cell microarray chips can be handled in the same way as for conventional microscopy with Giemsa staining for the staining of malaria-infected erythrocytes in the microchambers.

### Detection of malaria-infected erythrocytes on a cell microarray chip

For tight confinement of erythrocytes in the microchambers, erythrocytes in RPMI 1640 medium at a hematocrit of 0.25%, 0.5%, 0.75%, or 1.0% were dispersed on a cell microarray chip, followed by 10 min standing to allow the erythrocytes to settle down into the microchambers under gravitational force. The microchambers were then examined by light microscopy.

For analysis of cultured malaria-infected erythrocytes on a cell microarray chip, an appropriate volume of a purified malaria-uninfected erythrocyte suspension in RPMI 1640 medium was added to a malaria culture (0.4 to 1.0% parasitemia) to give 0.0001%–0.01% parasitemia and a 0.75% hematocrit, this step being performed within 5 min. An aliquot of a 5 mM stock solution of SYTO 59 (Life Technologies, Co., CA), which is a nuclear-specific fluorescence dye (Ex: 622 nm, Em: 645 nm), was added to each erythrocyte suspension to give a final concentration of 1 µM [17]. The erythrocyte suspension was dispersed manually on the cell microarray chip using a pipette, followed by 10 min standing, to allow the erythrocytes to settle down into the microchambers under gravitational force, and then nuclear staining with SYTO 59. Then excess cells on the chip surface were removed by gentle washing with RPMI 1640 medium. The microarray chip was scanned with a confocal laser-based fluorescence microarray scanner, CRBIO IIe (Hitachi Software Engineering Co., Ltd., Tokyo, Japan). This system exhibits resolution of up to 2.5 µm and a sensitivity of <0.1 fluorescent molecule/µm^2^, and is fitted with filters with emission wavelengths of 535 and 585 nm. The fluorescence intensity of individual erythrocytes was determined with DNASIS Array version 2.1 software (Hitachi Software Enginnering Co., Ltd.), and the erythrocytes that exhibited fluorescence intensities of two times above and ten times below that of uninfected erythrocytes were taken to be malaria-positive ones. The presence of malaria parasites in the fluorescence-positive erythorocytes was confirmed by Giemsa staining.

### Preparation of erythrocytes from whole blood and analysis on a cell microarray chip

For the preparation of erythrocytes from human whole blood without malaria infection, 20 µl of whole blood was mixed with 10 ml of RPMI 1640 medium, and the mixture was passed through LeukoLOCK™ under gravitational force. The isolation of leukocytes was performed with this isolation filter in less than 1 min. The eluted portion was centrifuged at 1,000×*g* for 2 min, and the pellet was dispersed in 1.0 ml of RPMI 1640 medium to give an appropriate hematocrit. SYTO 59 was added to the erythrocyte suspension, and the mixture was subjected to analysis on a cell microarray chip as described above. The effect of leukocyte contamination on a cell microarray chip analysis was examined.

### Statistical analysis

The number of fluorescence-positive erythrocytes was determined for each parasitemia sample, respectively. Data are expressed as the means ± standard error for five different experiments.

## Results

### Dispersion of erythrocytes on a cell microarray chip

**Figure 3:**
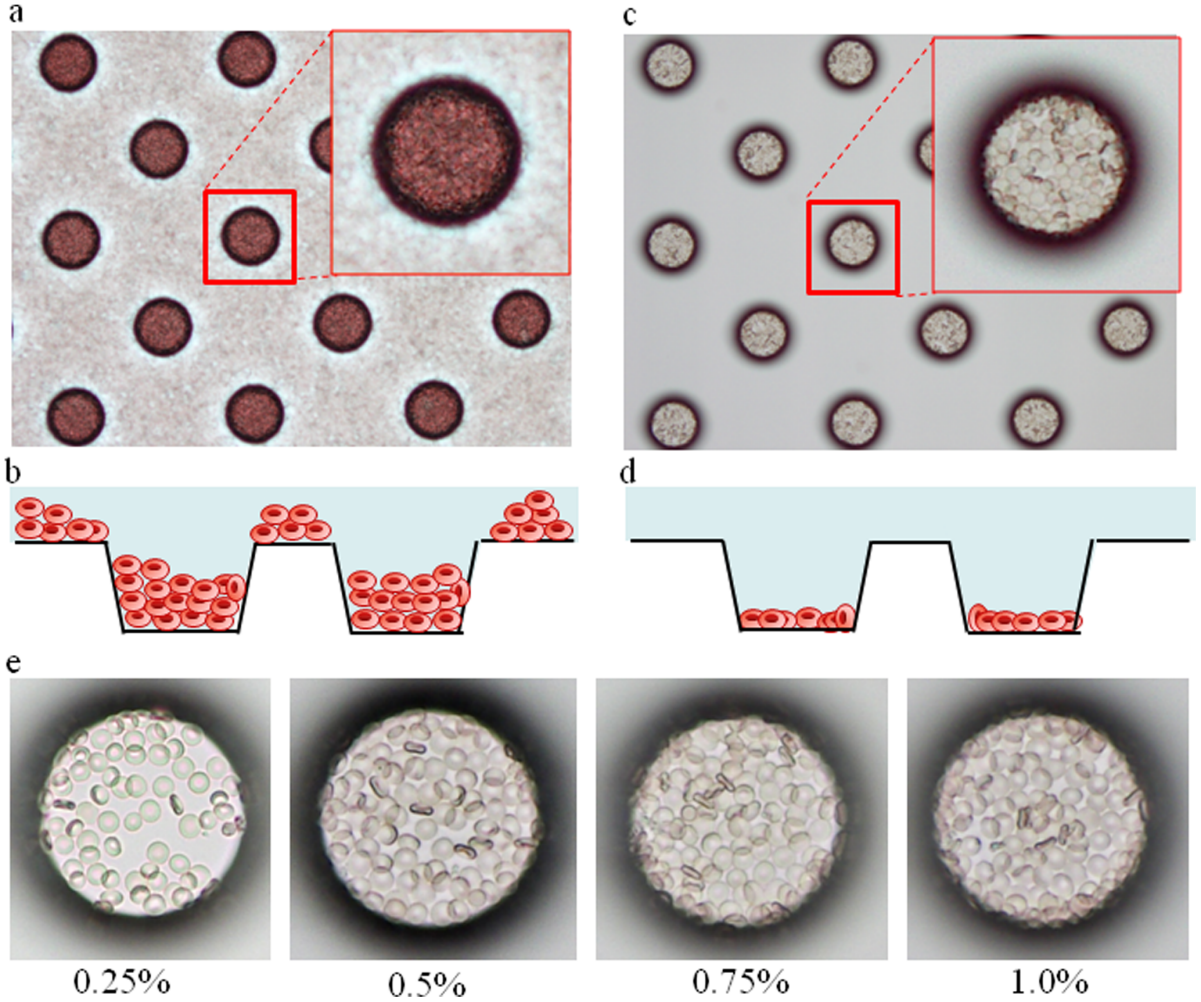
Dispersion of erythrocytes on a cell microarray chip and confinement in the microchambers. Photographic light microscopic images of erythrocytes on a cell microarray chip (**a**) before and (**c**) after washing of the chip surface. Schematic cross-section images of erythrocytes in microchambers (**b**) before and (**d**) after washing. After washing, the erythrocytes had formed a monolayer in the microchambers. (**e**) Real pictures of erythrocyte suspensions with hematocrits of 0.25, 0.5, 0.75 and 1.0 in microchambers after washing, respectively. The over 0.75% hematocrit samples show tight confinement of the erythrocytes in the microchambers.

To achieve the confinement of erythrocytes in the microchambers, the hydrophilicity on the microarray chip surface was optimized by means of reactive ion-etching exposure [14]. Eighty-seconds exposure gave appropriate hydrophilicity on the chip surface, and erythrocytes could settle in each microchamber (data not shown). For the formation of a monolayer of erythrocytes on the bottom surface of the microchambers after washing, the cell microarray chip was designed to have 105 µm diameter microchambers of 50 µm in depth and a cone-shaped frustum (Fig. 2d). The erythrocyte suspension is dropped on to a chip using a pipette, and then the erythrocytes settle down under gravitational force and adhere to the chip surface as multilayers (Fig. 3a, b). After washing of the chip surface using a pipette, erythrocytes only adhere to the bottom surface of each microchamber as a monolayer (Fig. 3c, d). It was observed that the number of erythrocytes confined in the microchambers depended on the erythrocyte concentration on the cell microarray chip, and erythrocytes were tightly confined with a hematocrit of over 0.75% (Fig. 3e). The number of confined erythrocytes was determined to be 130±6 (mean ± standard error) per microchamber (n = 30) with a hematocrit of over 0.75% (Fig. 3e). So, over 2,700,000 erythrocytes are dispersed as a monolayer in the microchambers on a microarray chip with a 0.75% hematocrit sample.

### Detection of malaria-infected erythrocytes on a cell microarray chip

**Figure 4:**
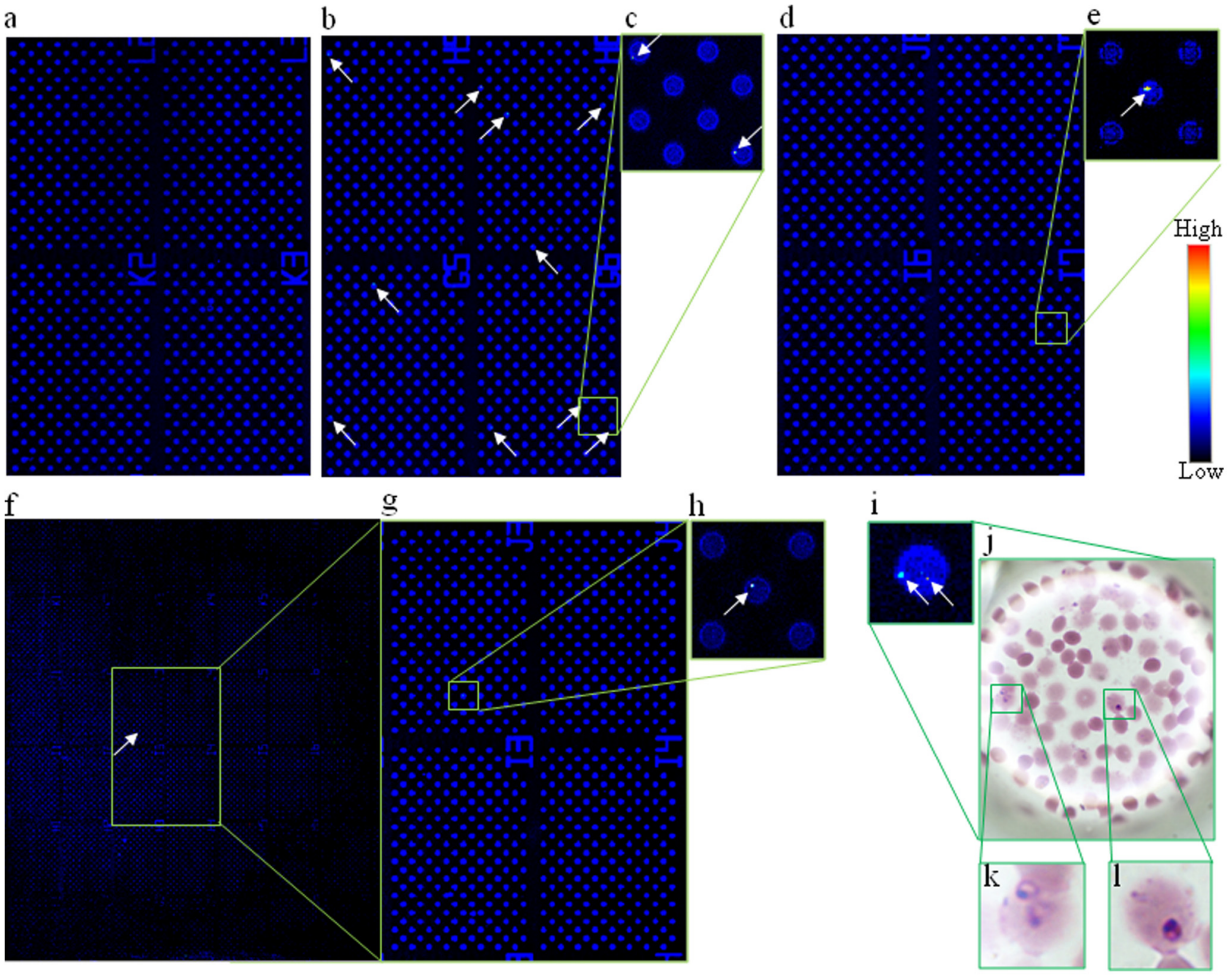
Detection of malaria-infected erythrocytes using a cell microarray chip. (**a–i**) Scanned images of malaria-infected erythrocytes on a microarray chip obtained with a microarray scanner. (**a**) Negative control (uninfected erythrocytes). (**b, d, f**) Malaria-infected erythrocytes (0.01, 0.001, and 0.0001%) were scanned in 4, 4, and 42 clusters on the microarray chip, respectively. (**c, e, g, h**) Magnified views of the boxed regions. (**i**) Microarray scanning images of malaria-infected erythrocytes. (**j, k, l**) Giemsa staining results for the cell microarray chip. (**k, l**) Two malaria-infected erythrocytes were observed in the microchamber on Giemsa staining.

Malaria-infected erythrocytes in SYTO 59 for staining of malaria parasite nuclei were dispersed on a cell microarray chip and then scanning images were obtained (Fig. 4a-h). Fluorescence-positive erythrocytes were not observed among erythrocytes used a negative control (Fig. 4a). Fluorescence-positive erythrocytes were observed in the microchambers with 0.01%, 0.001%, and 0.0001% parasitemia (Fig. 4b-h), the total numbers of fluorescence-positive erythrocytes from independent dilution experiments being 273.0±6.2, 35.6±2.9, and 4.0±0.3 (n = 5) in the whole microchamber area, as determined with DNASIS Array software, respectively. The percentage of parasitemia was determined using the following formula: [(number of fluorescence-positive erythrocytes/2,700,000 erythrocytes) ×100]. The percentages of parasitemia were calculated to be 0.010±0.0003%, 0.0013±0.0001%, and 0.00015±0.00001%, respectively. These calculated parasitemia levels were well consistent with the practical levels. To confirm the presence of malaria parasites in fluorescence-positive erythrocytes, Giemsa staining of the cell microarray chip was performed. High magnification images of microchambers containing fluorescence-positive erythrocytes were obtained (Fig. 4i). As shown in Fig. 4j-l, the presence of malaria parasites in the fluorescence-positive erythrocytes was confirmed.

### Discrimination of leukocytes and malaria-infected erythrocytes

**Figure 5:**
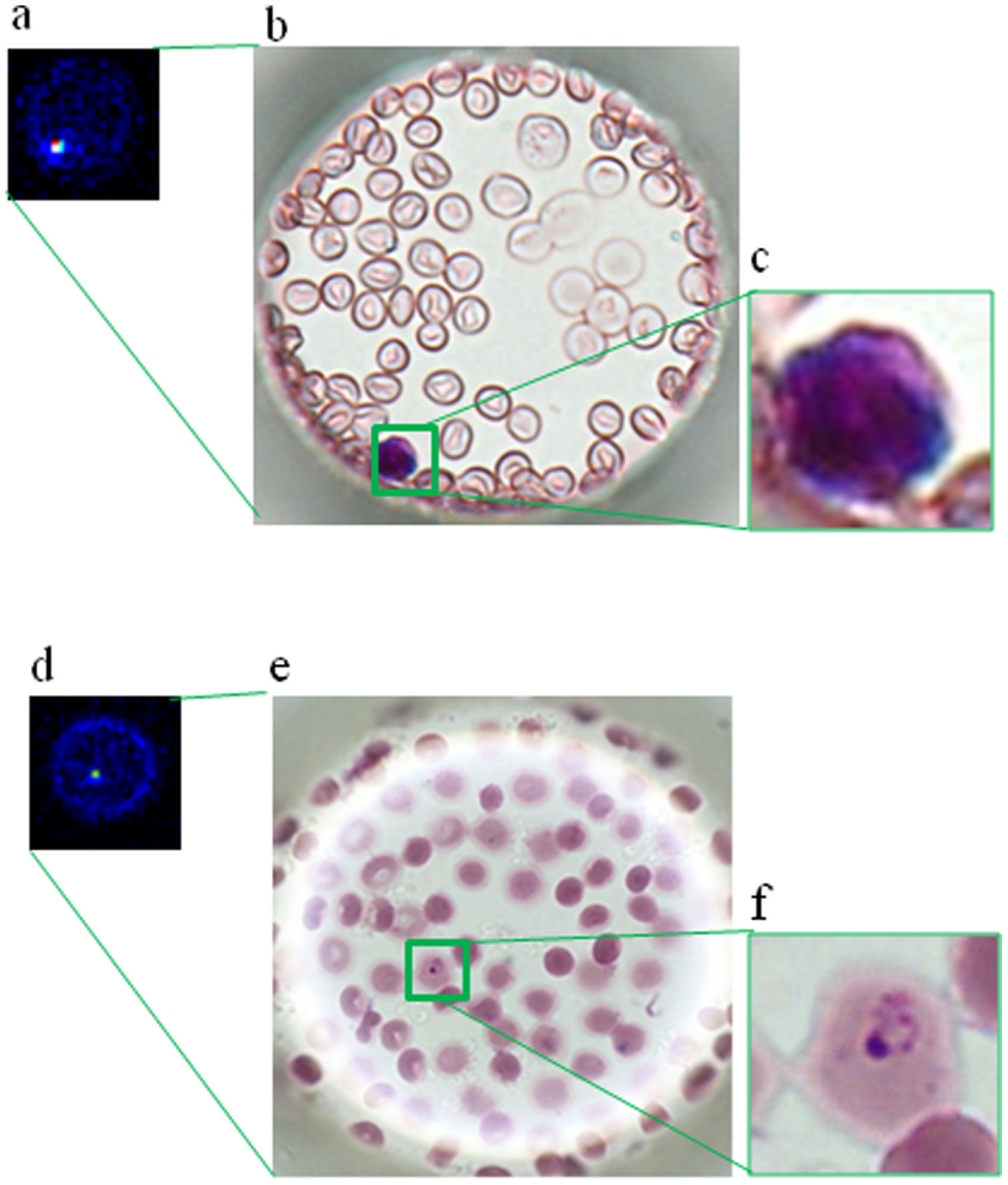
Discrimination of leukocytes and malaria-infected erythrocytes on a cell microarray chip. (**a**) Scanned image of leukocytes on a microarray chip obtained with a microarray scanner. (**b**) Leukocytes were identified by Giemsa staining. (**c**) Magnified view of the boxed region. (**d**) Microarray scanning image of malaria-infected erythrocytes. (**e**) Malaria-infected erythrocytes were confirmed by Giemsa staining after microarray scanning. (**f**) Magnified view of the boxed region.

To examine the possibility of contamination by leukocytes of purufied erythrocytes from whole blood, purified erythrocytes stained with SYTO 59 were analyzed on a cell microarray chip as described above. Similar accommodation of erythrocytes with tight confinement and monolayer formation in the microchambers using a malaria culture was observed on analysis of erythrocytes purified from whole blood (Fig. 5b), the number of confined erythrocytes being determined to be 127±5 (n = 30) per microchamber. Thus, there was no siginificant difference in the number of confined erythrocytes in the microchambers between whole blood and a malaria culture. Fluoresecence-positive leukocytes stained with SYTO 59 in the microchambers are shown in Fig. 5a, the fluorescence intensity of leukocytes is apparently higher than that of malaria parasites (Fig. 5d), and leukocytes can be easily distinguished from malaria-infected erythrocytes on the basis of the difference in fluorescence intensity. Furthermore, examination of contamination by leukocytes (Fig. 5b, c) and the presence of malaria-infected erythrocytes (Fig. 5e, f) in a malaria culture could be performed by Giemsa staining after microarray scanning. The total number of fluorescence-positive leukocytes was 0.6±0.4 (n = 5) in the whole microchamber area. This means the number of contaminating leukocytes was less than 1 on a cell microarray chip.

## Discussion

Our cell microarray chip exhibits 10–100 times higher sensitivity than that of light microscopy with Giemsa staining, and only 15 min is required for the detection of malaria parasites in purified erythrocytes. Although other more advanced methods based on flow cytometry or real-time PCR have been developed, the detection limit is only 0.003% for the flow cytometry measurement for parasitemia in malaria cultures with nuclear staining with YOYO-1 [10]. Flow cytometry analysis for the detection of malaria-infected erythrocytes is performed to evaluate the efficacy of antimalarial drugs [11]. It requires the fixation of cells with glutaraldehyde, RNase treatment, and staining with YOYO-1 in a 96-well plate format. Using a cell microarray chip for evaluation of the efficacy of antimalarial drugs, more sensitive and high throughput evaluation is anticipated. Furthermore, single cell observation can be performed using a cell microarray chip as opposed to population average studies on flow cytometry analysis. In real-time PCR, highly sensitive detection of malaria parasites (0.00001%) is performed, but several hours are required for the detection of malaria parasites [9], [12]. Although it is not possible to determine which of the different *Plasmodium* species of human is present in a positive sample with a cell microarray chip, high accuracy was obtained because only malaria-infected erythrocytes among mono-layered erythrocytes were targeted and detected with a confocal fluorescence laser scanning system. Erythrocytes for malaria cultures were prepared by centrifugation and a leukocyte isolation filter, and there is little possibility of contamination by means of leukocytes or cell debris of the sample for the cell microarray chip. The contamination risk is very low because we never found leukocytes or cell debris among over 100 fluorescence-positive erythrocytes on this cell microarray chip using a malaria culture. Using purified erythrocytes from human whole blood for cell microarray chip analysis, less than one leukocyte per 2,700,000 purified erythrocytes from whole blood was observed. If there is contamination by leukocytes in the erythrocyte suspension, it is relatively easy to distinguish malaria-infected erythrocytes and leukocytes by comparing their fluorescence intensities. The nuclei condensability in leukocytes is apparently greater than that in malaria parasites [18]. With this microarray scanner, the fluorescence intensity of malaria-infected erythrocytes is two times above and ten times below the fluorerscence intensity of uninfected erythrocytes. The fluorescence intensity of nuclei in leukocytes was ten times above that in uninfected erythrocytes, as shown in Fig. 5. Furthermore, Giemsa staining could be performed to confirm the presence of leukocytes after microarray scanning (Fig. 5b, c). There is very low contamination by leukocytes and no effect of platelets on erythrocyte binding in the microchambers. This may be due to the use of 500 times diluted whole blood for the leukocyte isolation filter and 50 times diluted eryhtrocytes for the cell microarray chip analysis. False positives can also be eliminated by Giemsa staining of the cell microarray chip after microarray scanning, as shown in Fig. 4i-l, and by determining the presence or absence of malaria-infected erythrocytes in the addressed microchamber as to fluorescence-positivity. Thus, a definitive diagnosis can be made at the single cell level.

Prompt and accurate diagnosis of malaria is a key factor for preventing contagion expansion. It has been reported that the median incubation period in nonimmune individuals (time from sporozoite incubation to development of symptoms) is 11 days (range, 6 to 14 days) [19]. Conventional light microscopy with Giemsa staining or immunochromatography is usually employed for malaria diagnosis after the appearance of symptoms including fever. Even in this case, there is a possibility of pass over or false positives because of the low sensitivity and/or low specificity. Another conventional method, PCR-based assaying, is more sensitive and specific than microscopic examination and immunochromatography for malaria diagnosis. However, PCR-based assaying is not suitable for the first screening of malaria because it involves complicated technical handling and is time-consuming. It was reported by Cheng *et al.* [20] that the multiplication rate of *P. falciparum* is 11.9-fold per 48 hours. So, at least 2–4 days earlier detection of malaria is expected with our cell microarray chip than with conventional microscopic examination because of the 10 to 100 times higher sensitivity. Such diagnosis may lead to the detection of malaria before the appearance of some symptoms. Although the cost of a cell microarray chip and the dye is less than US$ 1.0, the cost is US$ 40 for the isolation of leukocytes with LeukoLOCK™. Furthermore, a confocal laser-based fluorescence microarray scanner is a precision machine and expensive, about US$ 100,000. A cell microarray chip is suitable at room temperature, but fluorescence dye SYTO 59 must be stored at −20°C until use. So, this method is not useful in a field setting. It is recommended that the treatment response should be assessed by a daily parasite count until clearance of all trophozoites is achieved [17]. But the limitations of the conventional techniques for detecting *Plasmodium falciparum* infection cause serious difficulty in the identification and monitoring of malaria episodes [21]. For malaria therapeutic monitoring, detection with minimal operating steps is necessary, and high-accuracy, speed and easy operation are expected. Although blood from a malaria patient was not analyzed in the present study, our cell microarray chip system maybe suitable for malaria therapeutic monitoring because of the high sensivity and extremely low contamination by leukocytes. The incidence of cases of malaria from developing countries has risen, because of increasing in global travel and the migration of people from areas where malaria is endemic [19]. Therefore, the cell microarray chip system also has the potential for detecting malaria as a first screening system with good accuracy, for example, in the case of the necessity of performing early diagnosis of large numbers of people at an airport and/or a seaport for shoreline quarantine.
